# Complex Immune Contextures Characterise Malignant Peritoneal Mesothelioma: Loss of Adaptive Immunological Signature in the More Aggressive Histological Types

**DOI:** 10.1155/2018/5804230

**Published:** 2018-10-29

**Authors:** Marcella Tazzari, Silvia Brich, Alessandra Tuccitto, Fabio Bozzi, Valeria Beretta, Rosalin D. Spagnuolo, Tiziana Negri, Silvia Stacchiotti, Marcello Deraco, Dario Baratti, Chiara Camisaschi, Antonello Villa, Barbara Vergani, Licia Rivoltini, Silvana Pilotti, Chiara Castelli

**Affiliations:** ^1^Unit of Immunotherapy of Human Tumours, Department of Experimental Oncology and Molecular Medicine, Fondazione IRCCS Istituto Nazionale dei Tumori, Milan, Italy; ^2^Laboratory of Experimental Molecular Pathology, Department of Diagnostic Pathology and Laboratory Medicine, Fondazione IRCCS Istituto Nazionale dei Tumori, Milan, Italy; ^3^MOSE-DEA, University of Trieste, Trieste, Italy; ^4^Adult Mesenchymal Tumour and Rare Cancer Medical Oncology Unit, Cancer Medicine Department, Fondazione IRCCS Istituto Nazionale dei Tumori, Milan, Italy; ^5^Department of Surgery, Fondazione IRCCS Istituto Nazionale dei Tumori, Milan, Italy; ^6^Consorzio MIA, Microscopy and Image Analysis, University of Milan Bicocca, Monza, Italy

## Abstract

Malignant peritoneal mesothelioma (MpM), arising in the setting of local inflammation, is a rare aggressive tumour with a poor prognosis and limited therapeutic options. The three major MpM histological variants, epithelioid (E-MpMs), biphasic, and sarcomatoid MpMs (S-MpMs), are characterised by an increased aggressiveness and enhanced levels of EZH2 expression. To investigate the MpM immune contexture along the spectrum of MpM histotypes, an extended in situ analysis was performed on a series of 14 cases. Tumour-infiltrating immune cells and their functionality were assessed by immunohistochemistry, immunofluorescence, qRT-PCR, and flow cytometry analysis. MpMs are featured by a complex immune landscape modulated along the spectrum of MpM variants. Tumour-infiltrating T cells and evidence for pre-existing antitumour immunity are mainly confined to E-MpMs. However, Th1-related immunological features are progressively impaired in the more aggressive forms of E-MpMs and completely lost in S-MpM. Concomitantly, E-MpMs show also signs of active immune suppression, such as the occurrence of Tregs and Bregs and the expression of the immune checkpoint inhibitory molecules PD1 and PDL1. This study enriches the rising rationale for immunotherapy in MpM and points to the E-MpMs as the most immune-sensitive MpM histotypes, but it also suggests that synergistic interventions aimed at modifying the tumour microenvironment (TME) should be considered to make immunotherapy beneficial for these patients.

## 1. Introduction

Malignant peritoneal mesothelioma (MpM) arises from the mesothelial surface of the peritoneum. It is a rare aggressive cancer with an incidence of approximately 1 per 1000000, and it represents about 10–15% of all mesotheliomas [1]. Its major histological types include epithelioid (E-MpMs), biphasic, and sarcomatoid MpMs (S-MpMs). E-MpM is the least aggressive form but nevertheless has a sombre prognosis.

As in the case of pleural malignant mesotheliomas (MMs), the MpM aetiology includes asbestos exposure, which not only induces genotoxic carcinogenesis in mesothelioma cells but also directly activates an inflammatory autocrine pathway that recruits activated immune cells to the tumour site [2]. The complex interactions taking place in the tumour microenvironment (TME) give the tumour an aggressive evolution within a setting of an unresolved, long-lasting inflammation and immunosuppression [3]. Like pleural MMs, at least at the beginning of their development or in their less advanced/aggressive form, MpMs can be expected to show marked immune infiltration [4, 5] but, although recent findings have demonstrated the presence of CD3^+^ T cells and inflammatory cytokines in MpM ascites [6, 7], to the best of our knowledge, the immune contexture of MpMs has not been finely characterised yet.

We have recently suggested that the spectrum of the morphologic variants of MpMs may be governed by a process of mesenchymal epithelial reverse transition/epithelial mesenchymal transition (MErT/EMT) and demonstrated that EZH2, a member of the polycomb repressive complex 2 (PRC2), expression increases from E-MpMs to progressed (Pro) and high-grade/undifferentiated (HG) E-MpMs and S-MpMs [8]. It is worth noting that, in this continuum, Pro-E-MpMs represent an intermediate state.

EZH2 expressed by tumour cells has been reported to subvert the cytokine milieu, limit the recruitment of Th1 effectors, and ultimately favour immunoevasion [9, 10]. Thus, using the same series of surgical samples for which detailed molecular and morphological data are available [8], we extended the study to host components in order to provide a comprehensive view of the immune landscape of MpMs, including the expression of the immune checkpoint inhibitors (ICIs) PD1 and PDL1. Our data indicate that the immune contexture differs among the MpM variants with the Th1-related features present in the E-MpM variants but progressively weakened in the more aggressive forms of the E-MpM histotypes and completely lost in S-MpM, subtypes displaying enhanced expression of the transcriptional repressor EZH2.

## 2. Materials and Methods

### 2.1. Patient Samples

The case material consisted of formalin-fixed, paraffin-embedded (FFPE) surgical samples obtained from 14 previously untreated patients. The diagnoses, which were made on the basis of morphological and immunophenotypical criteria (calretinin, WT1, and cytokeratin 5/6) [11], as well as podoplanin as proposed in the WHO classification [12], were nine E-MpMs (no. 1–9, Table 1), two Pro-E-MpMs (no. 10-11, Table 1), one HG-E-MpM (no. 12, Table 1), and two S-MpMs (no. 13-14, Table 1).

The study was approved by the Independent Ethics Committee of the Fondazione IRCCS Istituto Nazionale dei Tumori di Milano (INT-MI). All of the patients whose biological samples were included in the study gave their written consent to donate the tissue remaining after their diagnostic procedures had been completed to the INT-MI.

### 2.2. Immunohistochemistry (IHC)

Representative 2 *μ*m sections of samples of all of the cases were selected and phenotyped. The antibodies and experimental conditions used to detect the expression of EZH2, CD3, CD8, CD4, Tbet, granzyme B (GZMB), Foxp3, PD1, PDL1, CD56, CD20, HLA class I, CD14, CD68, CD163, CD209, CD21, and VEGFR2 are shown in Supplementary Table S1. The slides were developed using 3,3-diaminobenzidine.

Evaluation of all IHC stains was always supervised and scored by a pathologist (S.P.). The scores were assigned semiquantitatively on a 0–3 scale, as follows 0 = no staining, 0.5 = occasional, 1 = low, 2 = intermediate, and 3 = high. T cell phenotype/functional markers (CD4, CD8, GZMB, PD1, Foxp3, and T-bet), macrophage markers (CD14, CD68, CD163, and CD209), CD20, CD56, HLA class I, and EZH2 assessments were performed by the pathologist, while intratumoural T lymphocyte infiltration (CD3 and CD8) was also analysed and scored using the “positive pixel count” algorithm of Aperio ImageScope (Version 12.1, Leica Biosystems, Wetzlar, Germany) after scanning of the whole tumour section. CD3^+^ and CD8^+^ T cell density was calculated as the number of positive pixels/*μ*m^2^. Scores given by the pathologist and Aperio scores correlated significantly (Spearman *r* = 0.95*p* value <0.0001 (^∗∗∗^) for CD3 and CD8; see Supplementary Figure S1).

### 2.3. Immunofluorescence (IF) and Confocal Analysis

The samples were analysed by means of confocal microscopy using a Radiance 2100 microscope (Bio-Rad Laboratories, Hercules, California, USA) equipped with a krypton/argon laser and red laser diode. The antibodies used are shown in Supplementary Table S1. The nuclei were stained with Toto-3 (Thermo Fisher Scientific, Waltham, MA, USA).

### 2.4. RNA Extraction, Reverse Transcription, and Real-Time Quantitative Reverse Transcription PCR

RNA was extracted from FFPE materials using MasterPure RNA Purification Kit (Illumina, San Diego, California, USA) following the manufacturer's instructions and quantified using a picodrop device. One thousand nanograms of RNA was used for cDNA synthesis. The cDNA was generated from 2.5 *μ*g of purified total RNA taken from each sample using a high-capacity cDNA reverse transcription kit (Thermo Fisher Scientific) in accordance with the manufacturer's instructions. The reverse transcription polymerase chain reaction (RT-PCR) was carried out using a GeneAmp PCR System 9700 instrument (Thermo Fisher Scientific) and the following settings: 25°C for 10 min and 60°C for 120 min. The cDNA was then preamplified using a TaqMan® PreAmp Master Mix Kit (Thermo Fisher Scientific) by combining 150 ng cDNA with TaqMan® PreAmp Master Mix (Thermo Fisher Scientific) and pooling the TaqMan® gene expression assays (Thermo Fisher Scientific) at a final concentration of 0.2x in accordance with the manufacturer's instructions. The real-time quantitative reverse transcription PCR (qRT-PCR) assays were run in the ABI 7900HT instrument (Thermo Fisher Scientific) with standard qRT-PCR settings: 50°C for 2 min, 95°C for 10 min, and 40 cycles of 95°C for 15 s and 60°C for 1 min. The data were analysed using SDS 2.2.2 software (Thermo Fisher Scientific) and are expressed as the mean and SD of the 2^−ΔCt^ values (ΔCt = Ct_target genes_–Ct_B2M_) of two technical replicates. The TaqMan® gene expression assays used in this work are reported in Supplementary Table S2.

### 2.5. Flow Cytometry

The flow cytometry analysis was made using thawed single-cell tumour suspension (patient no. 11, Table 1) obtained after collagenase digestion of fresh tumour material. Bregs were identified using anti-CD19 BV510, anti-CD5 APC, anti-CD38 PERCPCy5.5, anti-CD45 APCH7, anti-GZMB FITC (all from BD Biosciences, Franklin Lakes, New Jersey, USA), and anti-IL10 PE (BioLegend, San Diego, CA, USA). The cells were stained for cell surface markers after blocking nonspecific antibody binding to the Fc receptors using the FcR Blocking Reagent (Miltenyi, Bergisch Gladbach, Germany), fixed and permeabilised with Cytofix/Cytoperm buffer (BD Biosciences), and stained with the intracellular markers (IL10 and GZMB). Production of IL10 and GZMB was measured after overnight stimulation in the presence or absence of LPS (10 *μ*g/mL), PMA (50 ng/mL), and ionomycin (1 *μ*g/mL). GolgiStop (0.7 *μ*L/mL) was added after one-and-a-half hours' stimulation. Dead cells were identified using the LIVE-DEAD® Fixable Violet Dead Cell Stain Kit (Thermo Fisher Scientific) according to the manufacturer's instructions and excluded from the analysis. Data were acquired by flow cytometry (Gallios™, Beckman Coulter, Brea, California, USA), and analysis was done by Kaluza® Software (Beckman Coulter).

## 3. Results

### 3.1. MpM Variants Have Unique T Cell Landscapes

In the E-MpMs, H&E staining evidenced the presence of inflammatory infiltrating immune cells localised around the fibrovascular septa and near the tumour area (Supplementary Figure S2). Moreover, in the majority of E-MpMs, the large tumour nodules were surrounded by extranodal lymphoid structures (ELSs) arranged in a rosary-like pattern (data not shown). The infiltrating immune component was less evident, and ELSs were mainly virtually absent in HG-E-MPM and S-MpMs (data not shown). To define the nature of the immune infiltrating cells and the ELSs in the MpM variants, the whole series of cases was extensively analysed by means of IHC.

IHC analysis showed that CD3^+^-infiltrating cells, which included both CD4^+^ and CD8^+^, predominantly characterised E-MpMs. CD4^+^ and CD8^+^ T lymphocytes were clearly detectable in eight of the nine E-MpMs and in both Pro-E-MpMs. No or relatively few CD3^+^ cells were present in HG-E-MpM and in one of the two S-MpMs. The expression profile of the whole MpM series is reported in Table 1.

**Figure 1:**
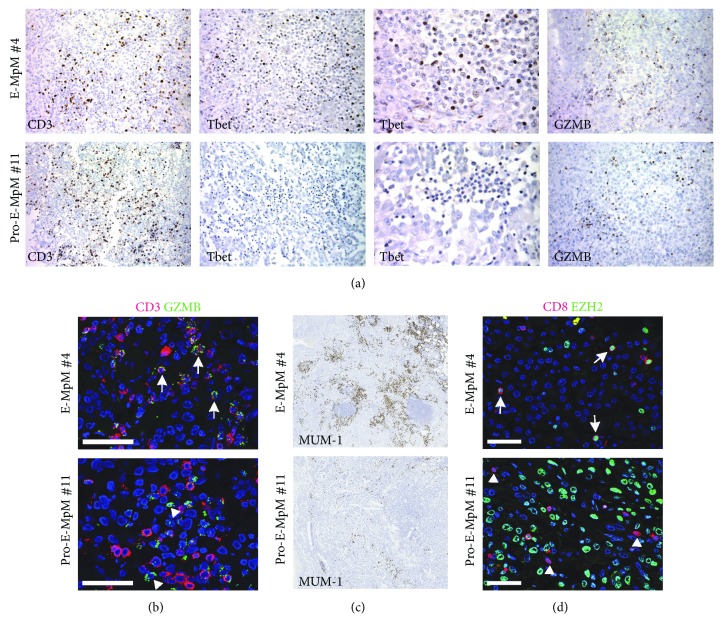
T cell infiltration and functional status across MpM variants. (a) Representative IHC images of CD3, Tbet, and granzyme B (GZMB) in an E-MpM (no. 4) and Pro-E-MpM (no. 11). Lower and the higher magnification for Tbet was reported. (b) Representative immunofluorescence (IF) staining and confocal analysis of the coexpression of GZMB (green) and CD3 (red) in an E-MpM (no. 4) and Pro-E-MpM (no. 11). The large majority of the cells in E-MpMs were double-positive (white arrow), whereas Pro-E-MpM no. 11 had a number of cells that were only GZMB-positive (white triangle). (c) Representative IHC images of MUM-1 in an E-MpM (no. 4) and Pro-E-MpM (no. 11). (d) IF staining and confocal analysis of the coexpression of CD8 (red) and EZH2 (green). Double-positive cells (white arrow) were detected in E-MpM (no. 7) whereas the majority of CD8^+^ cells in the Pro-E-MpM (no. 11) were single-positive (white triangle). The large green nuclei are tumour cells expressing EZH2. Nuclei were stained with TOTO-3 (blue). Scale bars = 50 *μ*m.

In order to characterise the nature of the infiltrating T cells, the expression of the nuclear transcription factor Tbet (a specific marker of anti-tumour Th1-polarised T cells) and granzyme B (GZMB), which is specific for the cytolytic molecules expressed by activated cytotoxic CD8^+^ T and NK cells, was evaluated (Figure 1). Almost all of the E-MpM cases were Tbet- and GZMB-positive; only one case (no. 5) was negative for both markers (Table 1). Conversely, Pro-E-MpMs, HG-E-MpM, and S-MpMs (nos. 10–14, Table 1) did not show any Tbet staining and were also negative for GZMB, with the exception of the strongly positive Pro-E-MpM (no. 11, Table 1).  Figure 1(a) shows the IHC staining for CD3, Tbet, and GZMB of E-MpM no. 4 (representative of the pattern found in all of the E-MpMs) and Pro-E-MpM no. 11. IF staining and confocal analysis showed that the large majority of GZMB^+^ cells were also positive for CD3 in E-MpM (Figure 1(b), upper panel). Conversely, only a few CD3^+^GZMB^+^ cells were found in Pro-E-MpM which was instead enriched in cells positive for GZMB but negative for CD3 (Figure 1(b), lower panel). The nature of these cells remains to be precisely established; nevertheless, this Pro-E-MpM no. 11 displayed a high frequency of CD56^+^ immune component (Table 1), thus suggesting that the strong GZMB positivity might be attributable to innate NK cells.

The Th1 nature of the immune cells infiltrating MpMs was further investigated by assessing the expression of interferon regulatory factor 4 (IRF4, also called MUM-1) in the TME and by testing the EZH2 positivity of MpM-infiltrating CD8 cells by means of IF and confocal analysis. In T cells, EZH2 and MUM-1 positively regulate Tbet, promote the differentiation of cytotoxic GZMB^+^ CD8 T cells, and sustain a polarised Th1 response [13–17]. E-MpM was highly positive for MUM-1 (Figure 1(c), upper panel), and a large majority of the CD8^+^-infiltrating cells were positive for EZH2 (Figure 1(d), upper panel), thus further supporting the Th1 polarisation of T cells infiltrating this variant, whereas MUM-1 was barely detectable in the Pro-E-MpM (Figure 1(c), upper panel), which also had few, if any, CD8^+^EZH2^+^ cells (Figure 1(d), lower panel). In Pro-E-MpM, CD8^+^ cells were mainly EZH2-negative (Figure 1(d), lower panel) compatible with their nonactivated and not finally differentiated effector status [13, 18]. Taken together, these findings indicate that the inflammatory T cell profile segregates with E-MpMs and their progressive phase and that Th1-related features are progressively impaired in the more aggressive forms of Pro- or HG-E-MpMs and in S-MpMs which displayed enhanced positivity for EZH2 expression in their tumour cells (Table 1) [8]. Therefore, our data are in line with what was already reported for other solid tumours [9] and highlight in the TME of MpM an association between the loss of Th1-specific features and EZH2 expression in cancer cells.

### 3.2. Absence of a Fully Competent T Cell Effector Signature in E-MpMs, and Evidence of Ongoing Immune Suppression

**Figure 2:**
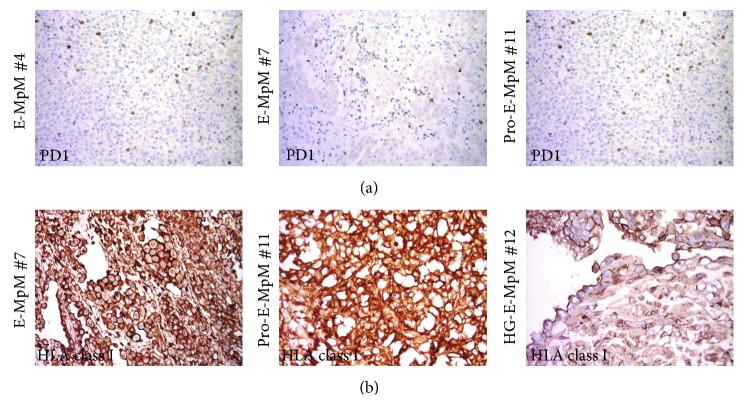
PD1 and HLA-class I expression in MpM. (a) Representative IHC images of intratumoural PD1 staining of E-MpMs. (b) IHC staining for HLA class-I. Strong membranous expression was detected in E-MpM no. 7 and Pro-E-MpM no. 11, and membranous decoration for HLA-class-I was retained in the residual epithelioid area of the HG-E-MpM (no. 12).

In order to define the differentiation status of the T cells infiltrating MpMs, PD1 expression was immunohistochemically assessed. As shown Figure 2(a) and summarised in Table 1, intratumoural PD1^+^ lymphocytes were detectable in four of the analysed E-MpMs, among those with greater CD8^+^ infiltration. PD1 is induced in T cells upon T cell receptor (TCR) stimulation, and so its expression bears witness to the presence of a subset of antigen-experienced T cells in E-MpMs. Together with the finding that E-MpMs, but not S-MpMs, expressed HLA class I molecules (Figure 2(b) and data not shown), and as in the case of melanoma [19], this suggests that the antigen-specific recognition of tumour cells may have occurred in E-MpMs and Pro-E-MpMs, as well as in HG-E-MpM (in which HLA class I positivity was still detectable in residual epithelioid areas) (Figure 2(b)).

**Figure 3:**
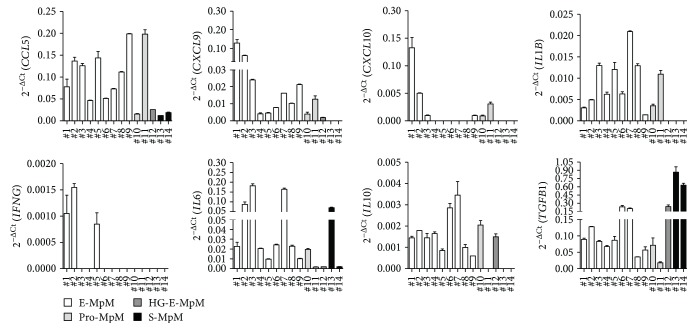
Gene expression of immune-related molecules in MpM tissues. The expression of *CCL5*, *CXCL9*, *CXCL10*, *IL1B*, *IFNG*, *IL6*, *IL10*, and *TGFB1* mRNAs was evaluated in the tissues of nine E-MpMs (nos. 1–9), two Pro E-MpMs (nos. 10-11), one HG-E-MpM (no. 12), and two S-MpMs (nos. 13-14). The data are expressed as the mean and SD of the 2^−ΔCt^ values (ΔCt = Ct_target genes_ − Ct_B2M_) of two replicates. mRNAs of *IL2*, *TNFA*, *IL4*, *IL5*, and *IL17B* genes were below the detection limit, and the corresponding CT values were undetermined.

However, as it is well known that PD1 upregulation in the TME is also associated with T cell exhaustion [20], we used qRT-PCR to investigate the expression of the chemokines/cytokines involved in T cell recruitment and effector functions in order to characterise further the tumour immune environment of the same case series. The *CCL5*, *CXCL9*, and *CXCL10* genes encoding for their specific chemokines regulating T cell trafficking in the TME were detectable in E-MpMs, albeit with different levels of expression, but hardly detectable or completely undetectable in HG-E-MpM and S-MpM, which is in line with the degree of lymphocyte infiltration detected by IHC (Figure 3 and Table 1). In terms of cytokines, *IL1B* was expressed by all of the E-MpMs, and the Th1 antitumour cytokine *IFNG* was clearly expressed in three, but negative in E-MpM samples yet displaying a strong CD3 cell infiltration, as assessed by IHC analysis (samples no. 4 and 6–9, Table 1 and Figure 3). It is worth noting that there was no expression of *IL2* or *TNFA* in any of the CD3-infiltrated samples, thus indicating that the Th1 immune cells infiltrating E-MpMs were not fully activated and likely functionally impaired. The Th2 cytokines, *IL5* and *IL4*, were also not detectable, thus ruling out the possibility for the setting of a Th2-related microenvironment (data not shown). Unlike the antitumour cytokines, the *IL6*, *IL10*, and *TGFB1* genes, encoding for immunosuppressive factors, were expressed by all of the E-MpM cases, which suggest the presence of an immunosuppression network. Of note, *TGFB1* was highly expressed in S-MpMs (Figure 3). With S-MpMs being poorly enriched in T cells, TGFB1 should not be derived from suppressive immune T cells, but it might be transcribed directly by the tumour cells, consistently with the mesenchymal nature of S-MpMs. One of the S-MpMs (case no. 13, Figure 3) also expressed *IL6.* The presence of *IL6* and *TGFB1* creates a cytokine microenvironment potentially favouring Th17 conversion. However, none of the examined E- or S-MpM samples displayed detectable Th17 cytokine transcript (data not shown).

As *TGFB1* and *IL10* are mediators of the effector functions of Tregs and Bregs, we looked for these immune cell subsets in order to further explore the immune suppressive traits of E-MpMs. Immunostaining for Foxp3, the nuclear transcription factor indicative of immunosuppressive Tregs, clearly revealed Foxp3^+^ in four of the nine E-MpMs (no. 1, no. 4, no. 6, and no. 8, Table 1) and in the two Pro-E-MpMs. The high intratumoural expression of Foxp3 paralleled the presence of T cell infiltration, which was also associated with the absence of Tbet^+^ T lymphocytes in the latter cases (Table 1 and Supplementary Figure S3). IHC showed that intratumoural CD20^+^ B cells were occasionally detectable in all of the analysed cases (Table 1) but mainly compartmentalized in discrete lymphoid aggregates with features of ELSs (Supplementary Figure S4).

**Figure 4:**
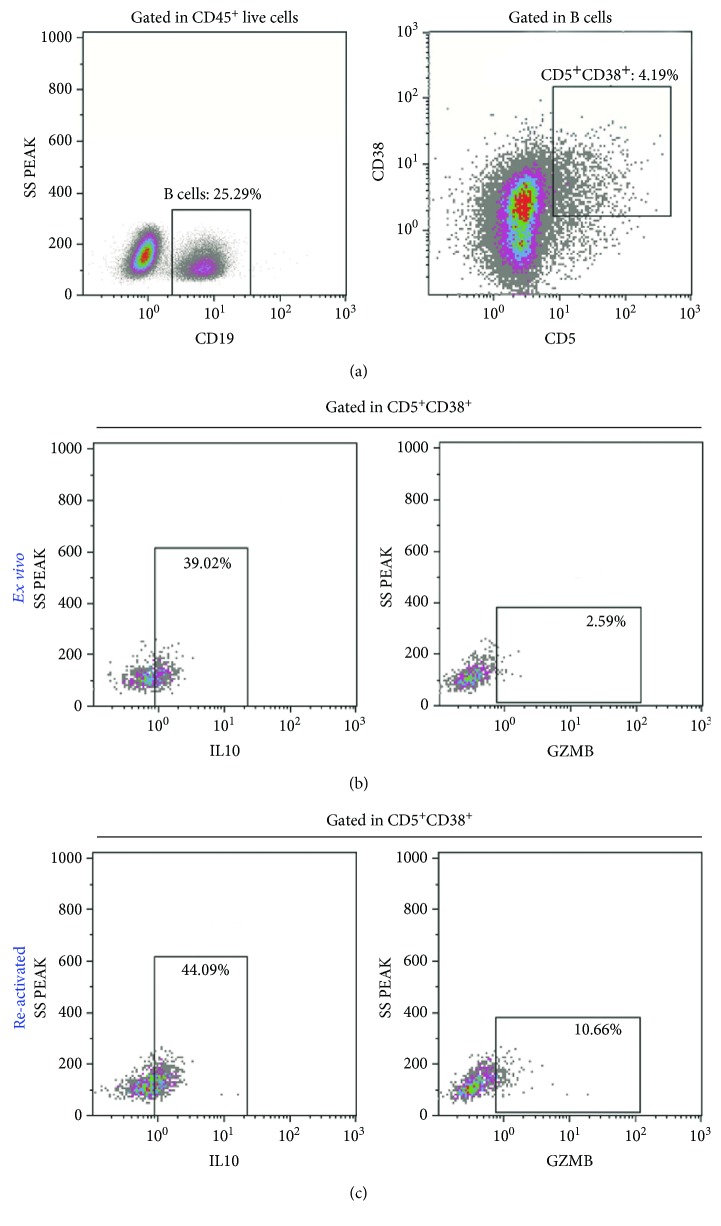
Presence of Bregs at the tumour site. Multiparametric flow cytometry analysis of tumour cell suspension of patient no. 11. Bregs were defined as CD19^+^CD5^+^CD38^+^ cells producing IL10 and GZMB. (a) Dot plots showing the gating strategy CD19^+^ B cells were defined inside live CD45^+^ cells, and the CD5^+^CD38^+^ double-positive population was identified among the live CD19^+^ B cells. The percentage of cells producing IL10 and GZMB was evaluated by intracellular staining in the CD5^+^CD38^+^ subpopulation among unstimulated cells (b) and cells stimulated overnight with LPS and PMA/ionomycin (c). The basal production of IL10 and GZMB is boosted by stimulation. The percentage of positive cells is shown in each plot.

As Bregs are defined by a complex phenotype that include simultaneous positivity for the markers CD19, CD38, CD5, GZMB, and IL10 [21], cells with this phenotype were investigated using multiparametric flow cytometry on the viable single-tumour cell suspension obtained from a fresh tumoural sample of patient no. 11. As shown in Figure 4(a), CD19-positive cells accounted for 25, 29% of tumour-infiltrating CD45^+^ cells. A small but detectable fraction (4.19%) of CD19^+^ B cells coexpressed CD5 and CD38 and was partially positive for IL10 and GZMB, a phenotype compatible with Bregs (Figure 4(b)). The fraction of CD19^+^CD5^+^CD38^+^ cells producing GZMB and IL10 increased upon activation (Figure 4(c)).

Altogether these data, achieved by different methodological approaches, concur to depict a complex immune landscape for E-MpMs. The TME of E-MpMs, while displaying an active immune profile sustained by the presence of HLA class I molecules, *CXCL9*, *CXCL10*, *IL1B*, and *IFNG* cytokines, it also carries immune-suppressive traits such as the presence of Foxp3^+^ Treg and *TGFB1* and *IL10* gene expression along with a fraction of B cells with a regulatory phenotype.

### 3.3. ELSs Characterise the E-MpM Microenvironment and Show Anti-tumour and Immunosuppressive Traits

The pattern of tumour-infiltrating lymphocyte (TIL) distribution identified dispersed lymphocytes or the presence of discrete lymphoid aggregates with features of ELSs mainly at the edges of large tumour nodules (Supplementary Figure S4A). ELSs are lymphoid structures that develop in nonlymphoid tissue and have an organisation similar to that of lymph nodes [22, 23]. In our samples, they showed discrete T (CD3^+^) and B (CD20^+^) cell areas, the first containing a CD21-positive follicular dendritic cell (FDC) network and the latter VEGFR2-positive endothelial venules, thus fulfilling the criteria for organised lymphoid structures (Supplementary Figure S4) [22, 23]. The ELSs closely associated with E-MpMs and Pro-E-MpMs (9 out of 11, Supplementary Table S3), but were absent from HG-E-MpM and S-MpMs (Supplementary Table S3). It is worth noting that in five of the nine E-MpMs, ELSs were also detected within the tumour nodules and, regardless of their intra- or peritumoural site, some had florid EZH2-labeled germinal centre (GC) B cells, thus indicating their active status (Supplementary Figure S4B and Supplementary Table S3) [24]. The immunological profile of the ELSs in the inflamed E-MpMs suggests their role in the recruitment and activation of specific T cells. Conversely, ELSs in Pro-E-MpMs had a less organised structure and were associated with smaller or burnt-out EZH2-expressing GCs (Supplementary Figure S4C and Supplementary Table S3).

The expression of the two checkpoint inhibitory molecules PD1 and PDL1 was assessed in all of the tumours. No tumour cells expressing them were found. However, their coordinated expression was found in the host immune component, with positivity mainly occurring in the most immune infiltrated E-MpMs. Higher staining was detectable in the ELSs (Supplementary Table S3 and Supplementary Figure S5). Five of the nine E-MpMs (nos. 1, 4, 5, 6 and 7) and one of the two Pro-E-MpMs (no. 10) were positive for both markers, whereas the other Pro-E-MpM (no. 11), the HG-E-MpM, and S-MpMs were negative.

In addition to B and T cells, Foxp3^+^ cells were also clearly detectable in ELSs (Supplementary Table S3). This, together with the positivity for PDL1, which is known to have immunosuppressive functions and is instrumental in the induction and functional activation of tumour-induced Tregs, suggests that ELSs may play a dual role in E-MpMs: in addition to recruiting T cells from the circulation, they may also sustain the immunosuppressive traits of E-MpMs [25, 26].

### 3.4. Macrophage Distribution and Phenotype in MpM

**Figure 5:**
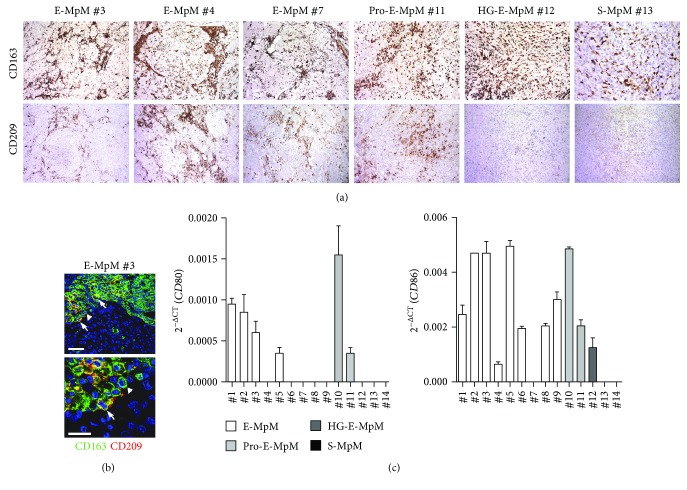
Analysis of infiltrating myeloid cells across MpM variants. (a) IHC images exemplifying CD163 and CD209 TAM immune profiles in E-MpM (nos. 3, 4, and 7), in Pro-E-MpM (no. 11), HG-E-MpM (no. 12), and S-MpM (no. 13). E-MpMs showed a fibrovascular pattern with immunolabeled TAMs mainly restricted around the septa which, in Pro-E-MpM no. 11, expand toward the central area. This compartmentalisation is lost in HG-E-MpM and S-MpM, both of which show a diffuse pattern with an increasing number of macrophages that are positive for CD163 but negative for CD209 immunostaining. (b) Representative IF staining and confocal analysis of CD209 (red) and CD163 (green) expression in one E-MpM (no. 3). A fraction of double-positive cells (white arrow) were detected, while the majority of cells were stained for CD163 (white triangle). Scale bars = 50 *μ*m and =5 *μ*m for the lower and higher magnification, respectively. (c) The expression of *CD80* and *CD86* mRNAs was evaluated in the tumour tissue of E-MpMs (nos. 1–9), Pro-E-MpMs (nos. 10-11), HG-E-MpM (no. 12), and S-MpMs (nos. 13-14). The data are mean and SD of the 2^−ΔCt^ values (ΔCt = Ct_target_ genes − Ct_B2M_) of two technical replicates. mRNAs of *IL12A* and *IL12B* genes were below the detection limit, and the corresponding CT values were undetermined.

In order to evaluate the presence and distribution of macrophages, we analysed in IHC a panel of known macrophage markers: CD14, CD68, and CD163. Similar positive immunolabeling for the three markers was present throughout the series, with an increasing number of decorated cells going from E-MpMs to HG-E-MpM and S-MpMs (Supplementary Table S4). The immunodecorated cells in the E-MpMs and Pro-E-MpMs had a fibrovascular pattern, whereas those in the HG-E-MpM and S-MpMs had a diffuse pattern: i.e., they were closely intermingled with tumoural cells in a patternless arrangement (Figure 5(a)).

In addition to the above myeloid-associated markers, we also assessed the expression of CD209, which is a documented M2 polarisation marker but is also involved in recognising pathogens and mediating phagocytosis in infectious diseases. Moreover, we have previously found an association between CD209^+^ tumour-associated macrophages (TAM) and an ongoing T cell response in a tumoural setting [27]. IHC analysis showed that CD209 expression segregated with E-MpMs and Pro-E-MpMs. Conversely, CD209 reactivity was undetectable in HG-E-MpM and S-MpM (Figure 5(a)) subtypes. Double IF staining and confocal analysis indicated that in E-MpM lesions, CD209 was coexpressed with CD163 (Figure 5(b)). This finding confirms that CD209^+^ myeloid cells are detectable in the TME enriched in T cells and provides additional evidence for the different nature of TAMs infiltrating E-MpMs versus those detected in the aggressive E-MpM and S-MpM variants. Gene expression data relating to the same cases as those analysed by IHC revealed the expression of the costimulatory molecules *CD80* and *CD86* only in E-MpMs and Pro-E-MpMs, but not in S-MpMs. HG-E-MpM was positive only for *CD86* (Figure 5(c)). As *CD80* and *CD86* are expressed by activated macrophages, we used qRT-PCR to check the expression of *IL12A* and *IL12B* genes encoding for the M1 cytokine IL12. No positivity of *IL12A* and *IL12B* genes was detected in any of the 14 analysed MpMs (data not shown), but *TGFB1* was present in all of the samples, and was clearly overexpressed in the S-MpMs (Figure 3).

Cumulatively, these findings suggest the presence of partially activated macrophages in E-MpMs, whereas those found in S-MpMs had likely a more frankly M2-like protumour phenotype.

### 3.5. Epithelial Cancer Cells with Features of Immune Cells in E-MpMs

**Figure 6:**
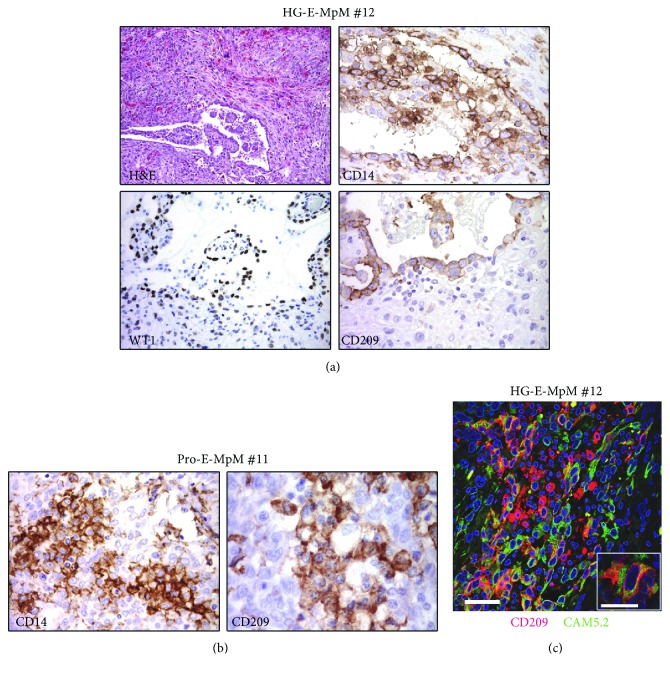
Epithelial cancer cells positive for myeloid related markers. (a) IHC images showing a residual area of HG-E-MpM (no. 12). The tumour cells were positive for WT1, and in the same area, cells with a frankly tumoural phenotype stained positive for the myeloid markers CD14 and CD209. (b) Small clusters of cells that are morphologically consistent with tumoural cells were positive for CD14 and CD209 in Pro-E-MpM no. 11. (c) Double IF immunostaining and confocal analysis of CD209 (red) and tumour-associated cytokeratin CAM5.2 (green) in HG-E-MpM (no. 12). Representative images of their coexpression in residual epithelioid featuring tumoural cells. Scale bars = 50 *μ*m and =5 *μ*m for the lower and higher magnification, respectively.

A unique feature of the higher grade of E-MpMs was the presence of randomly occurring small clusters of tumour cells expressing immune cell markers interspersed in the inflammatory immune component. There was morphologic evidence of this phenomenon in Pro-E-MpMs and HG-E-MpM no. 12 that still had retained small areas with epithelial features (Table 1 and Figure 6(a)). IHC analysis showed that these areas were positive for CD209, CD14, and the tumour marker WT1 (Figure 6(a)), whereas they were negative for the lineage-specific marker CD45 (data not shown). Cells with a frank tumoural phenotype and positive for CD14 and CD209 were also evidenced in Pro-E-MpM no. 11 (Figure 6(b)). IF and confocal analysis confirmed the coexpression of the CD209 myeloid marker and the cytokeratin CAM5.2 tumour marker (Figure 6(c)). These findings raise the possibility that, during the evolution toward the more aggressive forms, epithelial cells in E-MpMs acquire immune properties as a result of a transdifferentiation process, which is probably a mechanism underlying the evasion from an ongoing antitumour immune response [28].

## 4. Discussion

This study shows that the immune contexture of MpMs, which was investigated by means of multiple approaches including IHC, confocal analysis, qRT-PCR, and also flow cytometry in a digested surgical specimen, has a multifaceted profile that changes among the different MpM variants. Our analysis highlighted an evolution of the immune landscape that paralleled the MpM spectrum of progression. MpM is a rare aggressive cancer. Here, we analysed a total of 14 MpM cases: 9 belonging to epithelioid histotype which is the most common and 5 more progressed MpM cases, including the biphasic and sarcomatoid variants, which are very rare forms of MpM. Thus, while precisely describing the nature of immune infiltrate in the different forms of MpMs, our data should be further confirmed in future studies, especially for the setting of the most aggressive MpM histological variants.

Tumour-infiltrating lymphocytes were detectable throughout the E-MpM series and paralleled the coordinated expression of the CXCL9, CXCL10, and CCL5 chemokines acting in lymphocyte recruitment. However, Th1-polarised T cells were only found in E-MpMs. This Th1 signature, which was identified by means of positive staining for GZMB, Tbet, and MUM-1, and the presence of CD8^+^ cells expressing nuclear EZH2, was switched off in Pro-E-MpMs, HG-E-MpMs, and in S-MpMs. This lack of immune Th1 competence paralleled the increase in EZH2 positivity by cancer cells. Thus, our data suggest that EZH2 expression in tumour cells might contribute in shaping the nature of immune infiltration also in the setting of MpMs, as already proved in other solid tumours [9, 10].

The Th1 signature found in E-MpMs did not include a detectable *TNFA* or *IL2* gene transcription, suggesting that, despite their Th1 polarisation, the E-MpM-infiltrating T cells were probably dysfunctional or exhausted, which is in line with the detection of PD1 positivity in a fraction of E-MpMs. The impaired T cell immunity was associated with clear signs of immunosuppression and the presence of *IL10* and *TGFB1* cytokines in all of the E-MpM variants, albeit at different levels and with the clear prevalence of the first. Furthermore, immunophenotyping showed the presence of suppressive immunoregulatory Foxp3^+^ Tregs intratumourally or in ELSs, and CD20^+^ B cells. Multiparametric flow cytometry analysis complemented by activation assays showed that these B cells had a signature consistent with suppressive immunoregolatory Bregs: i.e., they were CD19^+^/CD38^+/^CD5^+^/GZMB^+^/IL10^+^ [29].

The enrichment of Bregs in MpMs may be due to the selective induction/recruitment by tumour cells. However, for physiological reasons, the peritoneum cavity is likely an immunologically protected environment in which inflammation needs to be under particularly tight control. Consistently, mouse peritoneal cavities are inhabited by anti-inflammatory lymphocytes specifically enriched in a subset of CD5^+^ B cells (also called CD5^+^ B1a cells) that express CD20, CD43, IgM^hi^, and IgD^lo^ and have tolerogenic functions [30, 31]. The existence of the human counterpart of murine B1a cells is still controversial, and no similar human data are currently available although various studies are being carried out (ClinicalTrials.gov Identifier: NCT03189316).

Mouse B1a and Bregs and human Bregs share a high capacity to produce IL10, a cytokine we found to be expressed in all of the E-MpM variants, and so it is entirely possible that the onset of IL10-driven immune suppression is an intrinsic characteristic of the peritoneum that protects the milieu against inflammation. The presence of the tumour is likely to amplify these conditions, thus leading to general and uncontrolled immune suppression.

The lymphocytic ELSs characterising the less aggressive E-MpMs were not found in the more aggressive variants or S-MpMs, and those present in one Pro-E-MPM had a nonfunctional organisation without germinal center B cells, thus further confirming the noninflamed nature of advanced MpMs.

Conversely, heavy myeloid cell infiltration was a trait shared by all of the MpM variants, although they could be differentiated on the basis of CD209 expression. We have previously described cells with a similar phenotype (e.g., CD14^+^, CD163^+^, and CD209^+^) as infiltrating post-imatinib-treated fibrosarcomatous dermatofibrosarcoma protuberans, and these same myeloid components have been also found in human renal carcinomas [27, 32]. In these settings and in MpMs, the presence of CD209^+^ macrophages is associated with heavy T cell infiltration, thus suggesting that these macrophage phenotypes may be the result of interplay between T cells and cells of the myeloid lineage, as has been found in mice [33]. The myeloid cells of E-MpMs likely had a more activated pattern than those of S-MpMs, as measured by the gene expression of the activation molecules *CD80* and *CD86* in the TME. Although human CD209^+^ macrophages have been described as having enhanced phagocytosis in the context of sepsis [34] and are thus probably associated with enhanced protective immunity, we did not detect any *IL12* mRNA expression (the key cytokine qualifying M1 antitumour macrophages) in E-MpMs. Further studies aimed at defining the role played by CD209^+^ macrophages in these tumours are warranted.

Intriguingly, myeloid-specific markers were also occasionally expressed in Pro-E-MpMs and HG-E-MpM by small scattered clusters of tumour cells showing immune cell-like differentiation: i.e., epithelial-immune cell-like transition (EIT) or epithelial-myeloid transition [28, 35]. This process leads to tumour cells acquiring phenotypic and functional features typical of the myeloid lineages, including a high degree of plasticity, migration capacity, tissue remodelling, and most importantly, immunologic competence in terms of immune evasion and suppression. EIT therefore seems to be a crucial step toward more aggressive E-MpMs in which transdifferentiation may be detected in Pro-E-MpMs and in the residual epithelioid foci of HG-E-MpMs, but not in fully mesenchymal S-MpMs. The mechanisms underlying the acquisition of macrophage traits by tumour cells are still unknown, but cell fusion and paracrine cellular interactions have been suggested as possible explanations [36].

None of the tumour cells in our samples expressed PDL1, which is in line with recently published data showing that PDL1 are rarely expressed in mesothelioma cells [37]. PD1-positive immune cells were found exclusively in E-MpMs, and they were located intratumoural and within ELSs in the large majority of cases. However, despite the small number of immunolabeled cells, there was a positive association between PDL1- and PD1-positive TILs and between PDL1 expression and the frequency of Foxp3-positive lymphocytes, which is in agreement with the finding that PDL1 can induce, enhance, and maintain the expression of the *FOXP3* gene in induced animal Tregs [25].

It is known that an exhausted immune response can be rescued through PD1/PDL1 antibodies provided that the proportion of activated PD1-positive lymphocytes is quite high and PDL1 is expressed by a sizeable number of host immune cells and tumour cells. On the basis of our analysis, among the PDL1 patterns proposed as indicators of the likelihood of response to the anti-PDL1 antibody [38], E-MpMs seem to fall into the “nonfunctional immune response” category. Conversely, the links with the MErT/EMT process and the stemness traits would place S-MpMs in the “noninflamed EMT/stem-like type” category [39]. Untreated or naive MpMs therefore do not seem to be promising targets for PD1/PDL1 inhibitors, but other checkpoint inhibitors may be active. The current immune-based therapies for peritoneal mesothelioma include the treatment with the anti-CTLA4 antibody tremelimumab, and a phase III, randomised trial is ongoing (Clinical Trial NCT01843374) [40].

In conclusion, we found that the MpM variants include different entities in terms of their immune contexture, and our data suggest that, as in other solid tumour, the nature of TME of MpMs might be linked to the epigenetic modifier EZH2. Among the MpM histotypes, only E-MpMs show evidence of pre-existing T cell tumour immunity and their retained HLA class I expression bears witness to the possible immune sensitivity of epithelial mesothelioma tumour cells. The expression of PDL1 and PD1 is detected in a subset of E-MpMs; nevertheless, the presence of a highly suppressive environment (which probably also reflects the immunologically protected peritoneal compartment) poses limits to the possible efficacy of immunotherapy. The landscape of S-MpMs is even more negative as there is no evidence for an activated lymphocyte infiltration (Supplementary Figure S6).

## 5. Conclusions

Our results enrich the knowledge on the immune cell composition of this rare and aggressive neoplasm and strongly indicate that in MpM interventions aimed at modifying the TME should be considered. The administration of drug silencing tumoural EZH2 (currently explored as monotherapy in the NTC022899195 clinical trial in malignant mesothelioma patients), given to favour lymphocyte recruitment and constrain immune suppression, might be an option to be tested in combination with immune-based approaches such as blockage of ICIs.

## Figures and Tables

**Table 1:** IHC characterisation of intratumoural lymphocytes in MpM variants.

Case	MpM variants	Intratumoural lymphocytes										
		Tumour cells	T cells							NK cells	B cells	Tumour cells
		EZH2	CD3^∗^^II^	CD8^∗^^II^	CD4	Tbet	GZMB	Foxp3	PD1	CD56	CD20	HLA class I^§^
No. 1	E-MpM	1	3	3	2	2	3	2	0.5	0	0.5	Positive
No. 2	E-MpM	1	0.5	0.5	0.5	0.5	0.5	0	0	0	0.5	Positive
No. 3	E-MpM	1	1	1	0.5	0.5	1	0.5	0	0.5	0.5	Positive
No. 4	E-MpM	1	2	2	2	1	2	2	0.5	1	0.5	Positive
No. 5	E-MpM	1	1	1	1	0	0	0.5	0	0	0.5	Positive
No. 6	E-MpM	1	2	2	2	2	1	1	0	0	1	Positive
No. 7	E-MpM	1	2	2	2	2	1.5	0.5	0.5	3	0.5	Positive
No. 8	E-MpM	1	2	2	1	2	2	1	0	1	0.5	Positive
No. 9	E-MpM	1	2	2	0.5	1	0.5	0.5	0	2	0.5	Positive
No. 10	Pro-E-MpM	2	1	1	2	0	0	1	0	0	0.5	Positive
No. 11	Pro-E-MpM	2	2	2	1	0	3	2	0.5	3	0.5	Positive
No. 12	HG-E-MpM	3	0.5	0.5	0	0	0	0	0	1	0.5^‡^	Negative^¶^
No. 13	S-MpM	2	1	1	0	0	0	1	0	0	0.5	Negative
No. 14	S-MpM	1	0	0	0	0	0	0	0	0	0	Negative

Note: E-MpM: epithelioid malignant peritoneal mesothelioma; Pro: progressed; HG: high-grade; S: sarcomatoid; GZMB: granzyme B. ^∗^Scored by the pathologist (S.P.). The scores were assigned semiquantitatively on a 0–3 scale, as follows: 0 = no staining, 0.5 = occasional, 1 = low, 2 = intermediate, and 3 = high. ^†^Automated scoring using Aperio ScanScope. ^‡^In very small clusters. ^§^HLA class I was evaluated as negative or positive without assigning a semiquantitative score. ^¶^With the exception of small residual epithelioid cells with tumour foci. ^II^Scores given by the pathologist and Aperio scores correlated significantly; see Supplementary Figure S1.

## Data Availability

The immunohistochemistry, immunofluorescence, gene expression, and flow cytometry data used to support the findings of our study are included within the article. The immunohistochemistry data used to support the findings of our study are included within the supplementary information file.

## Supplementary Materials

Supplementary 1 Table S1: antibodies used for immunohistochemistry (IHC)/immunofluorescence (IF) analysis. Table S2: panel of TaqMan® assays used for gene expression analysis. Table S3: characterisation of ELSs. Table S4: IHC characterisation of intratumoural TAMs in MpM variants.

Supplementary 2 Figure S1: correlations between IHC scores given by the pathologist and Aperio ScanScope for CD3 and CD8.

Supplementary 3 Figure S2: distribution of inflammatory infiltrating immune cells by hematoxylin and eosin staining.

Supplementary 4 Figure S3: Foxp3 expression in MpM cases.

Supplementary 5 Figure S4: ELS characterisation by immunohistochemistry.

Supplementary 6 Figure S5: PD1 and PDL1 expression in ELSs.

Supplementary 7 Figure S6: a graphical summary of the immune contexture in MpM variants.
