# Supplementary material for: Complex Immune Contextures Characterise Malignant Peritoneal Mesothelioma: Loss of Adaptive Immunological Signature in the More Aggressive Histological Types
Source: J Immunol Res. 2018 Oct 29;2018:5804230. doi: 10.1155/2018/5804230 (PMC6231377; doi:10.1155/2018/5804230)
Supplement: Supplementary 1 — Table S1: antibodies used for immunohistochemistry (IHC)/immunofluorescence (IF) analysis. Table S2: panel of TaqMan® assays used for gene expression analysis. Table S3: characterisation of ELSs. Table S4: IHC characterisation of intratumoural TAMs in MpM variants. [file 5804230.f1.docx]

**SUPPLEMENTAL DATA**

**SUPPLEMENTARY FIGURES AND FIGURE LEGENDS**

**

**

**Supplementary Figure S1. Correlations between IHC scores given by the Pathologist and Aperio ScanScope for CD3 and CD8.** Spearman’s correlation analysis was performed for IHC quantification of CD3 and CD8 markers in the cohort of 14 MpMs. The scores were assigned semi-quantitatively by the Pathologist (S.P.) and were quantified using the “positive pixel count” algorithm of Aperio ImageScope (Version 12.1) after scanning of the whole tumour sections. The R values, Spearman’s correlation coefficient, and p-values are reported.

**
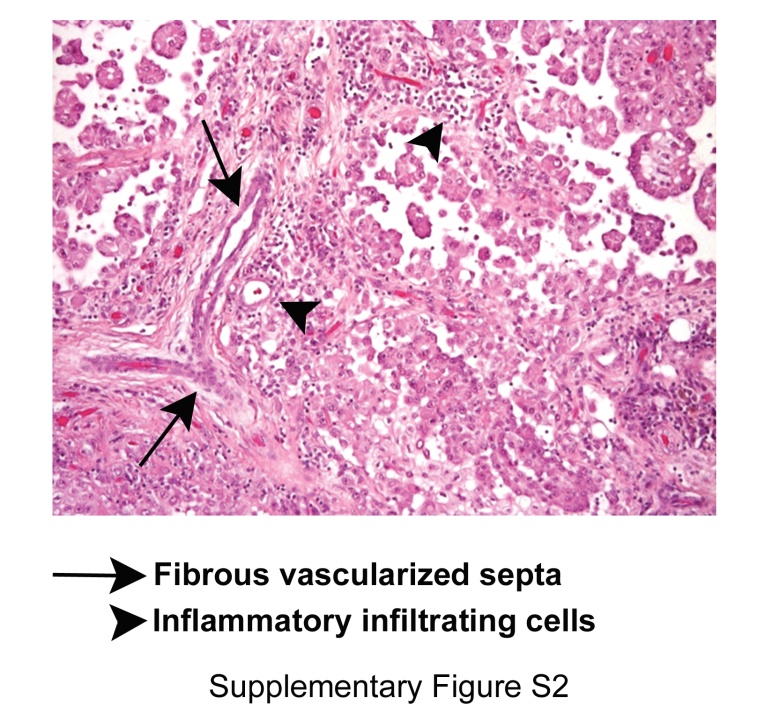
**

**Supplementary Figure S2. Distribution of inflammatory infiltrating immune cells by hematoxylin and eosin staining.** Low power view of hematoxylin and eosin stained samples showing the inflammatory infiltrating immune cells distributed in a fibrovascular-like pattern mainly restricted to the septa and relatively near to tumour area.

**
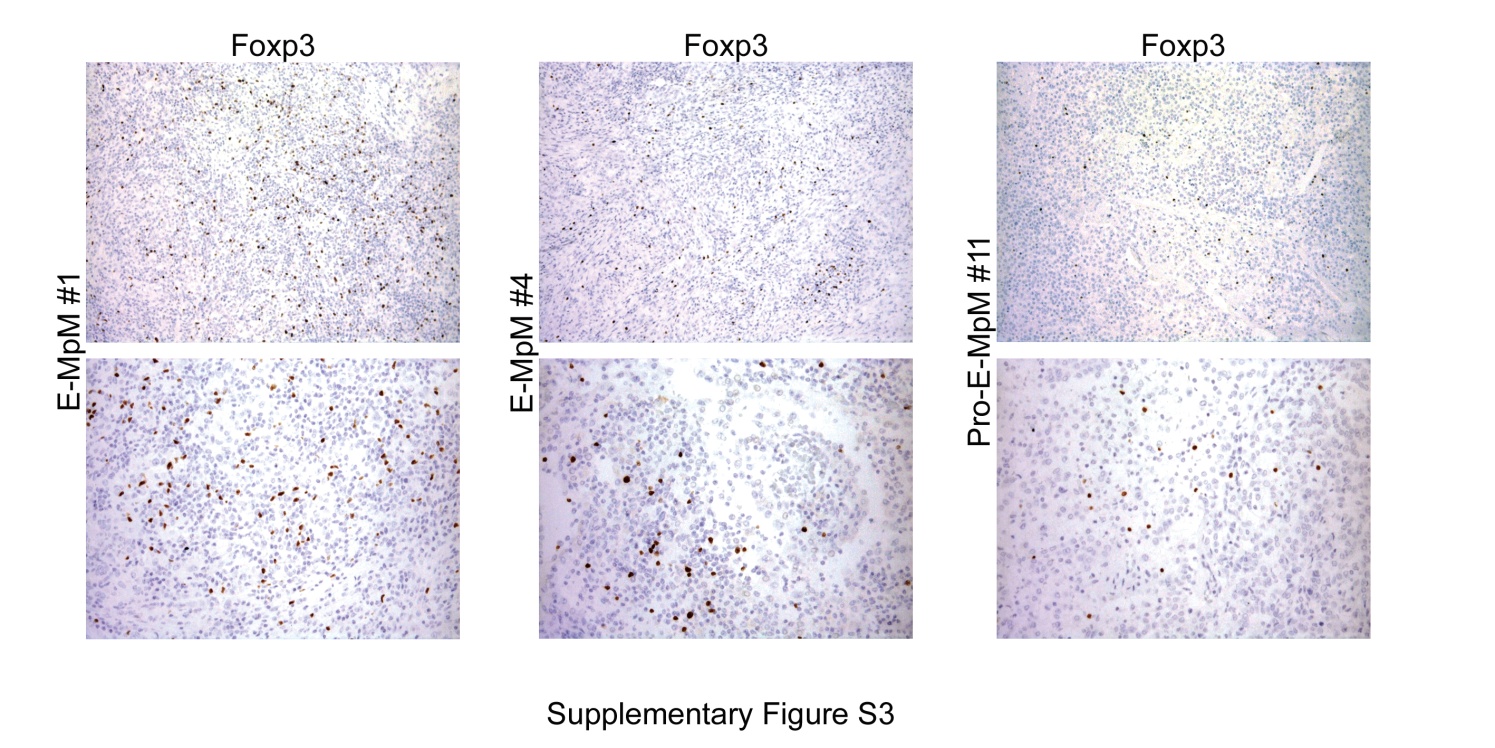
**

**Supplementary Figure S3. Foxp3 expression in MpM cases.** Image gallery showing Foxp3 immunolabeled intratumoural lymphocytes at low (upper row) and higher magnification (lower row). E-MpMs #1 and #4 were more enriched in positive cells than Pro-E-MpM #11.

**
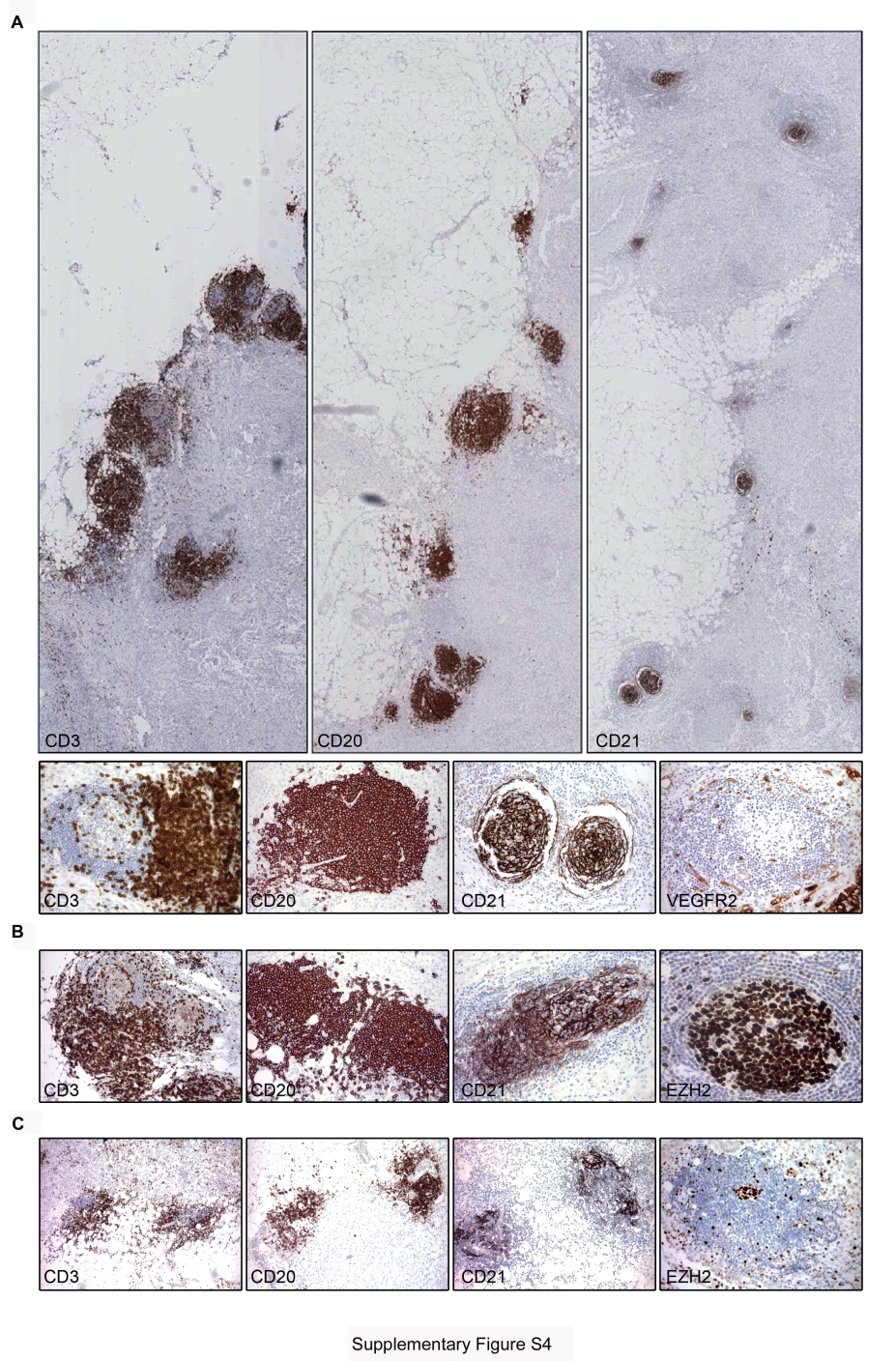
**

**Supplementary Figure S4. ELS characterisation by immunohistochemistry.** The whole-slide image and representative snapshots were acquired using a ScanScope XT/XT2 digital scanner (Aperio ePathology Solutions, Vista, CA, USA). **(A)** Upper panels: low power view showing CD3-, CD20- and CD21-decorated ELSs distributed in a rosary-like pattern at the edges of tumoural growth. Lower panels: ELSs showing discrete T (CD3) and B (CD20) areas, and a well-developed CD21 network in germinal centre (GC) and endothelial venules (VEGFR2) in the T cell area. **(B)**. Examples of florid ELSs with GCs enriched in CD21 and active EZH2-labeled B cells. **(C)** Examples of burnt out ELSs.

**
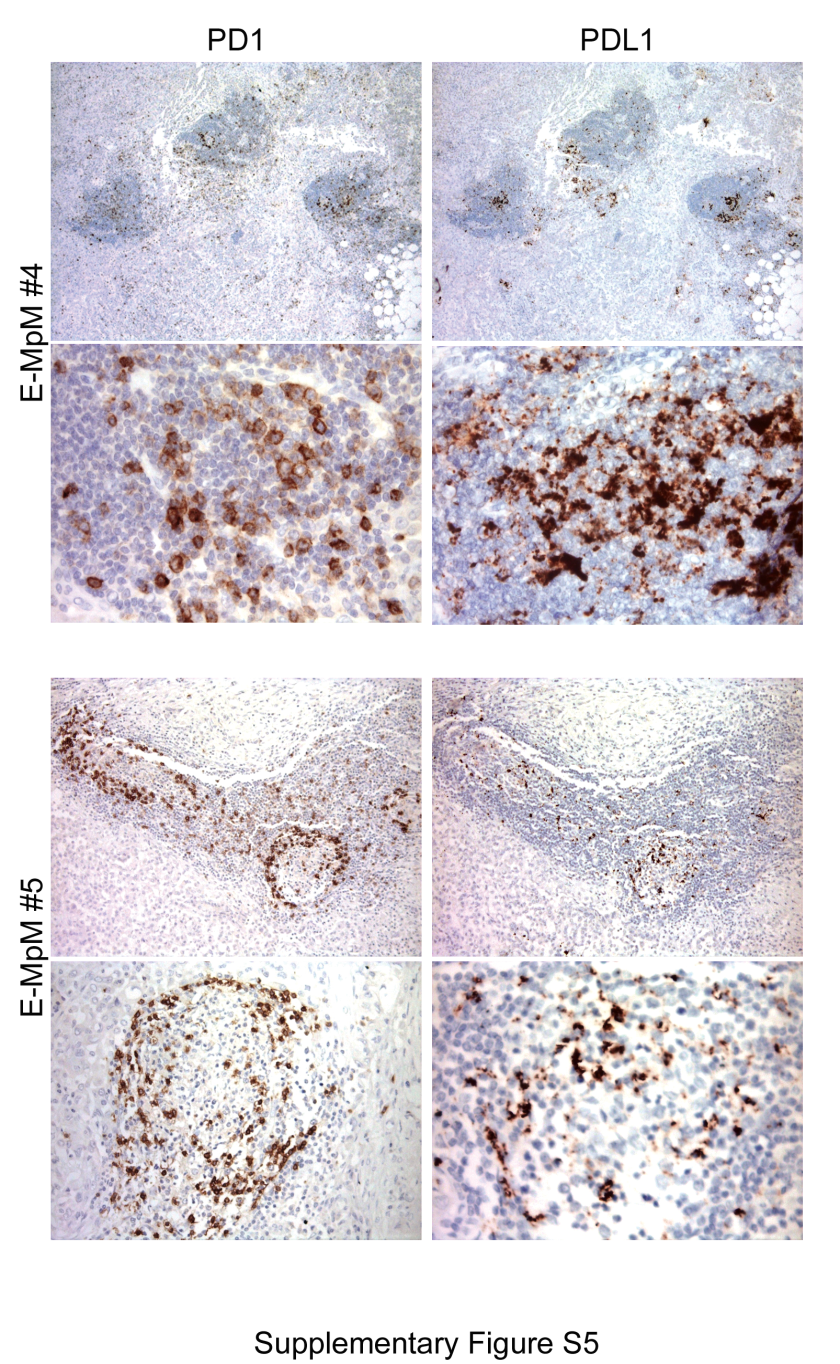
**

**Supplementary Figure S5. PD1 and PDL1 expression in ELSs.** Serial sections of E-MpM #4 and E-MpM #5 showing ELSs immunolabeled with PD1 and PDL1.

**
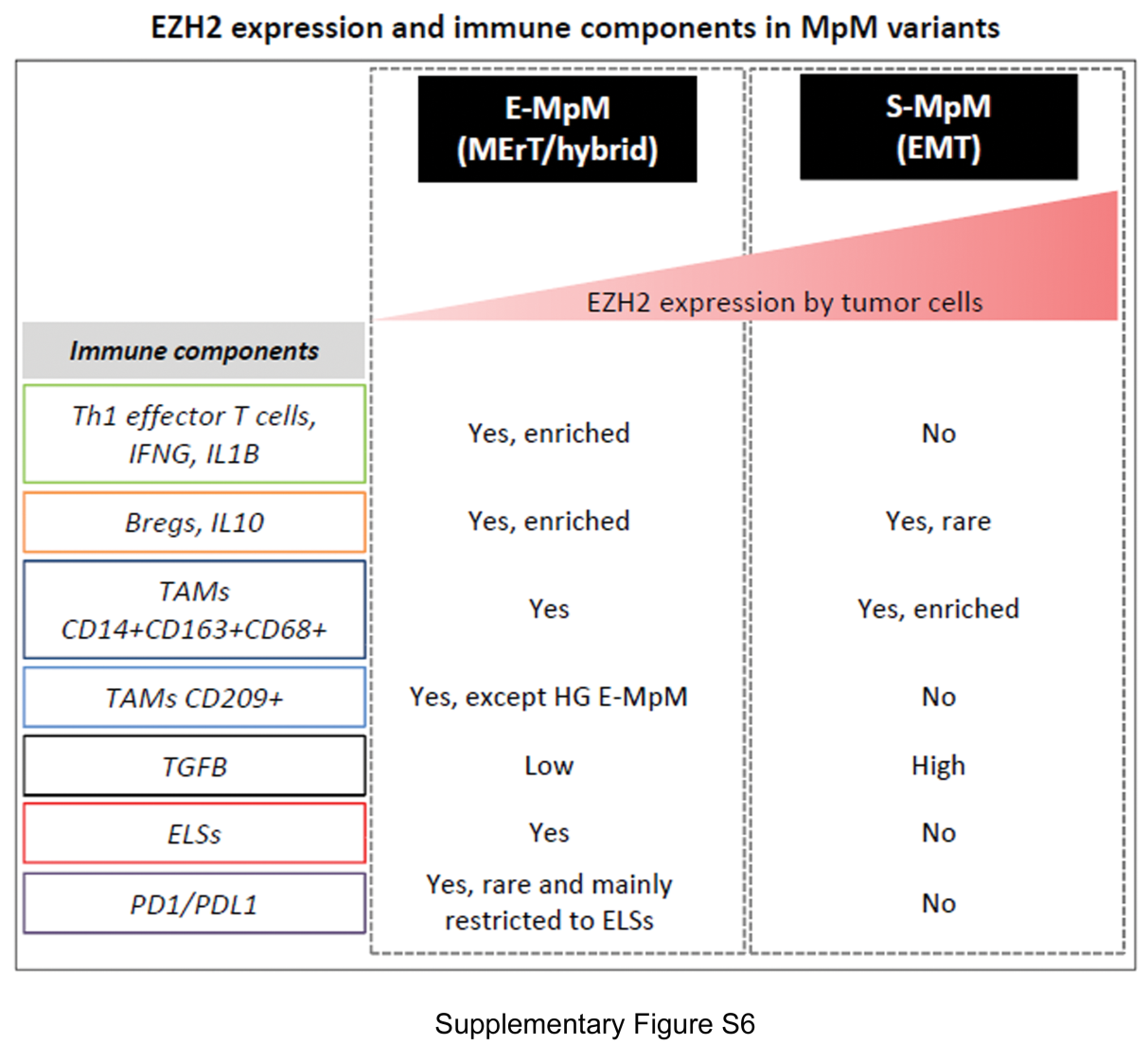
**

**Supplementary Figure S6. A graphical summary of the immune contexture in MpM variants.** The immune contexture and the expression of EZH2 are modulated along the spectrum of MpM variants. Tumour infiltrating T cells displaying Th1 features and the expression of targetable immune checkpoints PD1/PDL1 are confined to E-MpMs. However, E-MpMs are also infiltrated by immune suppressive cells. No evidence for T cell infiltration is detectable in S-MpMs which are highly positive for EZH2, display a mesenchymal phenotype and are strongly enriched in the myeloid CD163+ cells.

**Supplementary Table S1. Antibodies used for immunohistochemistry (IHC)/immunofluorescence (IF) analysis**

| **Antibody** | **Catalog number** | **Company** | **Clone** | **Isotype** | **Staining platform** | **Dilution** | **Antigen retrieval** | **Visualisation system** |
| --- | --- | --- | --- | --- | --- | --- | --- | --- |
| **EZH2** | 5246 | Cell Signaling | D2C9 | monoclonal rabbit | Ventana Benchmark Ultra | IHC:1:50  IF:1:5 | standard,CC1 buffer,optiviewDAB  EDTA buffer pH 8,  pressure cooker 20' at 110°C | OptiView DAB IHC Detection Kit Ventana Roche  anti-rabbit 488 Alexa Fluor® |
| **CD3** | A0452 | DAKO | /* | polyclonal rabbit | Manual staining | IHC:1:400  IF:40 | EDTA buffer pH 8,  pressure cooker 20' at 110°C | Ultravision Quanto Detection System HRP Thermo + Liquid DAB substrate chromogen System DAKO  anti rabbit 468 Alexa Fluor® |
| **CD8** | IR623 | DAKO | C8/144B | monoclonal mouse IgG1 | Manual staining | IHC:1:20  IF:1:5 | EDTA buffer pH 8,  pressure cooker 20' at 110°C | Ultravision Quanto Detection System HRP Thermo + Liquid DAB substrate chromogen System DAKO  anti-IgG1 568 Alexa Fluor® |
| **CD4** | MA5-12259 | Thermo Fisher Scientific | 4B12 | monoclonal mouse IgG1 | Manual staining | IHC:1:300 | EDTA buffer pH 8,  pressure cooker 20' at 110°C | Ultravision Quanto Detection System HRP Thermo + Liquid DAB substrate chromogen System DAKO |
| **Tbet** | sc-21003 | SantaCruz | H-210 | monoclonal mouse IgG1 | Manual staining | IHC:1:80  IF:1:10 | Citrate buffer pH 6,  pressure cooker 20' at 110°C | Ultravision Quanto Detection System HRP Thermo + Liquid DAB substrate chromogen System DAKO  anti-IgG1 568 Alexa Fluor® |
| **GZMB** | NCL-L-GRAN-B | Novocastra | 11F1 | monoclonal mouse IgG2a | Manual staining | IHC: 1:80  IF:10 | EDTA buffer pH 8,  pressure cooker 20' at 110°C | Ultravision Quanto Detection System HRP Thermo + Liquid DAB substrate chromogen System DAKO  anti-IgG2a 488 Alexa Fluor® |
| **Foxp3** | 560044 | BD | 259D/C7 | monoclonal mouse IgG1 | Manual staining | IHC:1:100 | EDTA buffer pH 8,  pressure cooker 15' at 110°C | Ultravision Quanto Detection System HRP Thermo + Liquid DAB substrate chromogen System DAKO |
| **PD1** | ACI 3137 AK | Biocare | NAT105 | monoclonal mouse IgG1 | Ventana Benchmark Ultra | IHC:1:100 | extended, CC1 buffer | OptiView DAB IHC Detection Kit +OptiView Amplification Kit Ventana Roche |
| **PDL1** | 740-4859 | Roche | SP142 | polyclonal rabbit | Ventana Benchmark Ultra | IHC:1:50 | standard, CC1 buffer | OptiView DAB IHC Detection Kit+OptiView Amplification Kit Ventana Roche |
| **CD56** | IR628 | Dako | 123C3 | monoclonal mouse IgG1 | Dako Autostainer Link 48 | IHC:1:400 | 30'high | EnVision FLEX +30'LinkerMouse DAKO Agilent |
| **CD20** | M0755 | DAKO | L26 | monoclonal mouse IgG2a | Manual staining | IHC:1:400 | EDTA buffer pH 8,  pressure cooker 20' at 110°C | Ultravision Quanto Detection System HRP Thermo + Liquid DAB substrate chromogen System DAKO |
| **HLA Class I** | ab70328 | Abcam | EMR8-5 | monoclonal mouse IgG1 | Manual staining | IHC:1:100 | Citrate buffer pH 6,  pressure cooker 15' at 110°C | Ultravision Quanto Detection System HRP Thermo + Liquid DAB substrate chromogen System DAKO |
| **CD14** | ab13335 | Abcam | EPR3653 | monoclonal rabbit | Ventana Benchmark Ultra | IHC:1:1000 | reducted,CC1 buffer | OptiView DAB IHC Detection Kit Ventana Roche |
| **CD163** | NCL-CD163 | Novocastra | 10D6 | monoclonal mouse IgG1 | Ventana Benchmark Ultra | IHC: 1:100  IF:10 | reducted,CC1 buffer | OptiView DAB IHC Detection Kit Ventana Roche  anti-IgG1 488 Alexa Fluor® |
| **CD68** | IR609 | DAKO | KP1 | monoclonal mouse IgG1 | Dako Autostainer Link 48 | IHC:1:3000 | 30'low | EnVision FLEX +30'LinkerMouse DAKO Agilent |
| **CD209** | 551249 | BD | DCN46 | monoclonal mouse IgG2b | Manual staining | IHC: 1:20  IF:1:5 | Citrate buffer pH 6,  pressure cooker 15' at 110°C | Ultravision Quanto Detection System HRP Thermo + Liquid DAB substrate chromogen System DAKO  anti-IgG2b 568 Alexa Fluor® |
| **CD21** | IR608 | DAKO | IF8 | monoclonal mouse IgG1 | Dako Autostainer Link 48 | IHC:1:50 | 30'low | EnVision FLEX +30'LinkerMouse DAKO Agilent |
| **VEFGR2** | 2479 | Cell Signaling | 55B11 | monoclonal rabbit | Ventana Benchmark Ultra | IHC:1:300 | extended,CC1 buffer | UltraView Universal DAB Detection Kit Ventana Roche |
| **CAM5.2** | 3652 | DAKO | EP17/EP30 | monoclonal rabbit | Manual staining | IF:1:100 | Citrate buffer pH 6,  pressure cooker 15' at 110°C | anti-rabbit 488 Alexa Fluor® |
| Note: *, not available. | | | | | | | | |

**Supplementary Table S2. Panel of TaqMan^®^ assays used for gene expression analysis**

| **Gene name** | **Gene symbol** | **RefSeq (NM)** | **Assay ID^*^** | **Amplicon length** | **Protein name** |
| --- | --- | --- | --- | --- | --- |
| **Beta-2-microglobulin** | ***B2M*** | NM_004048.2 | Hs99999907_m1 | 75 | Beta-2-microglobulin |
| **C-C motif chemokine ligand 5** | ***CCL5*** | NM_001278736.1  NM_002985.2 | Hs00982282_m1 | 70 | C-C motif chemokine 5 |
| **CD80 molecule** | ***CD80*** | NM_005191.3 | Hs00175478_m1 | 107 | T-lymphocyte activation antigen CD80 |
| **CD86 molecule** | ***CD86*** | NM_001206924.1  NM_001206925.1  NM_006889.4  NM_175862.4  NM_176892.1 | Hs01567026_m1 | 104 | T-lymphocyte activation antigen CD86 |
| **C-X-C motif chemokine ligand 9** | ***CXCL9*** | NM_002416.2 | Hs00171065_m1 | 60 | C-X-C motif chemokine 9 |
| **C-X-C motif chemokine ligand 10** | ***CXCL10*** | NM_001565 | Hs00171042_m1 | 98 | C-X-C motif chemokine 10 |
| **Interferon gamma** | ***IFNG*** | NM_000619 | Hs00989291_m1 | 73 | Interferon gamma |
| **Interleukin 1 beta** | ***IL1B*** | NM_000576.2 | Hs01555410_m1 | 91 | Interleukin-1 beta |
| **Interleukin 2** | ***IL2*** | NM_000586.3 | Hs00174114_m1 | 94 | Interleukin-2 |
| **Interleukin 4** | ***IL4*** | NM_000589.3  NM_172348.2 | Hs00174122_m1 | 70 | Interleukin-4 |
| **Interleukin 5** | ***IL5*** | NM_000879.2 | Hs01548712_g1 | 99 | Interleukin-5 |
| **Interleukin 6** | ***IL6*** | NM_000600.4 | Hs00985639_m1 | 66 | Interleukin-6 |
| **Interleukin 10** | ***IL10*** | NM_000572.2 | Hs00961622_m1 | 74 | Interleukin-10 |
| **Interleukin 12A** | ***IL12A*** | NM_000882.3 | Hs01073447_m1 | 52 | Interleukin-12A |
| **Interleukin 12B** | ***IL12B*** | NM_000879.2 | Hs01548712_g1 | 99 | Interleukin-12B |
| **Interleukin 17B** | ***IL17B*** | [NM_014443.2](http://www.ncbi.nlm.nih.gov/nuccore/NM_014443.2) | Hs00205368_m1 | 65 | Interleukin-17B |
| **Transforming growth factor beta 1** | ***TGFB1*** | NM_000660.6 | Hs00998133_m1 | 57 | Transforming growth factor beta-1 |
| **Tumour necrosis factor** | ***TNF*** | NM_000594.3 | Hs00174128_m1 | 80 | Tumour necrosis factor |
| Note: ^*^ **TaqMan^®^ Gene Expression Assays purchased from Thermo Fisher Scientific** | | | | | |

**Supplementary Table S3. Characterisation of ELSs**

| **Case** | **MpM variants** | **ELSs** | | | | | **ELS germinal centers** | |
| --- | --- | --- | --- | --- | --- | --- | --- | --- |
|  |  | **CD3** | **CD20** | **Foxp3** | **PD1*** | **PDL1^†^** | **CD21** | **EZH2** |
| **#1** | **E-MpM** | 0.5 | 1 | 2 | 1, Peritumoural and intratumoural | 1, Peritumoural and  ~20% intratumoural^‡^ | 2 | 1 |
| **#2** | **E-MpM** | 0 | 0 | 0.5 | 0 | 0 | 0 | 0 |
| **#3** | **E-MpM** | 3 | 3 | 1 | 0 | 0 | 0.5 | 1, Burnt out |
| **#4** | **E-MpM** | 3 | 3 | 2 | 1, Peritumoural | 1, Peritumoural ~30%^‡^ | 2 | 2 |
| **#5** | **E-MpM** | 3 | 3 | 2 | 1, Peritumoural | 1, Peritumoural ~10%^‡^ | 3 | 2 |
| **#6** | **E-MpM** | 3 | 3 | 2 | 1, Peritumoural | 1 Peritumoural | 2 | 2 |
| **#7** | **E-MpM** | 3 | 3 | 0.5 | 1, Intratumoural | 1, Intratumoural ~10%^‡^ | 2 | 2 |
| **#8** | **E-MpM** | 1 | 2 | 1 | 0 | 0 | 1 | 0 |
| **#9** | **E-MpM** | 0 | 0 | 0.5 | 0 | 0 | 0 | 0 |
| **#10** | **Pro-E-MpM** | 2 | 3 | 1 | 1, Peritumoural and intratumoural | 1, Peritumoural and intratumoural | 2 | 1 |
| **#11** | **Pro-E-MpM** | 3 | 3 | 2 | 0 | 0 | 2 | 1, Burnt out |
| **#12** | **HG-E-MpM** | No ELS | No ELS | No ELS | 0 | 0 | 0 | 0 |
| **#13** | **S-MpM** | No ELS | No ELS | No ELS | 0 | 0 | 0 | 0 |
| **#14** | **S-MpM** | No ELS | No ELS | No ELS | 0 | 0 | 0 | 0 |
| ELSs: extranodal lymphoid structures; E-MpM: epitheliod malignant peritoneal mesothelioma; Pro: progressed; HG: high-grade; S: sarcomatoid, *: in small clusters; expressed in lymphocyte-like cells; †: expressed in monocyte-/dendritic-like cells. The scores were assigned semi-quantitatively on a 0-3 scale, as follows: 0=no staining, 0.5=occasional, 1=low, 2=intermediate, 3=high. ‡: PDL1/CD3 ratio. For additional subpopulations see text and microphotograph. | | | | | | | | |

**Supplementary Table S4. IHC characterisation of intratumoural TAMs in MpM variants**

| **Case** | **MpM variants** | **Intratumoural TAMs** | | | |
| --- | --- | --- | --- | --- | --- |
|  |  | **CD14** | **CD68** | **CD163** | **CD209**^*^ |
| **#1** | **E-MpM** | 3 | 3 | 3 | 1 |
| **#2** | **E-MpM** | 1 | 1 | 1 | 0.5 |
| **#3** | **E-MpM** | 2 | 2 | 2 | 1 |
| **#4** | **E-MpM** | 3 | 3 | 3 | 1 |
| **#5** | **E-MpM** | 2 | 2 | 3 | 1 |
| **#6** | **E-MpM** | 2 | 2 | 2 | 2 |
| **#7** | **E-MpM** | 2 | 2 | 1 | 1 |
| **#8** | **E-MpM** | 0.5 | 1 | 1 | 0.5 |
| **#9** | **E-MpM** | 3 | 2 | 2 | 0.5 |
| **#10** | **Pro-E-MpM** | 2 | 1 | 2 | 0.5 |
| **#11** | **Pro-E-MpM** | 3 | 3 | 3 | 1^†^ |
| **#12** | **HG-E-MpM** | 3 | 3 | 3 | 0 |
| **#13** | **S-MpM** | 3 | 3 | 3 | 0 |
| **#14** | **S-MpM** | 2 | 3 | 2 | 0 |
| Note: E-MpM: epitheliod malignant peritoneal mesothelioma; Pro: progressed; HG: high-grade; S: sarcomatoid. TAMs: tumour-associated macrophages. The scores were assigned semi-quantitatively on a 0-3 scale, as follows: 0=no staining, 0.5=occasional, 1=low, 2=intermediate, 3=high. ^*^CD209 staining was evaluated in infiltrating inflammatory cells; ^†^The expression of CD209 was also detected in a defined epithelial tumour area in tumours #11 and #12. See Figure 6 and text for details. | | | | | |
