# Supplementary material for: Complex Immune Contextures Characterise Malignant Peritoneal Mesothelioma: Loss of Adaptive Immunological Signature in the More Aggressive Histological Types
Source: J Immunol Res. 2018 Oct 29;2018:5804230. doi: 10.1155/2018/5804230 (PMC6231377; doi:10.1155/2018/5804230)
Supplement: Supplementary 7 — Figure S6: a graphical summary of the immune contexture in MpM variants. [file 5804230.f7.pptx]

## Slide 1
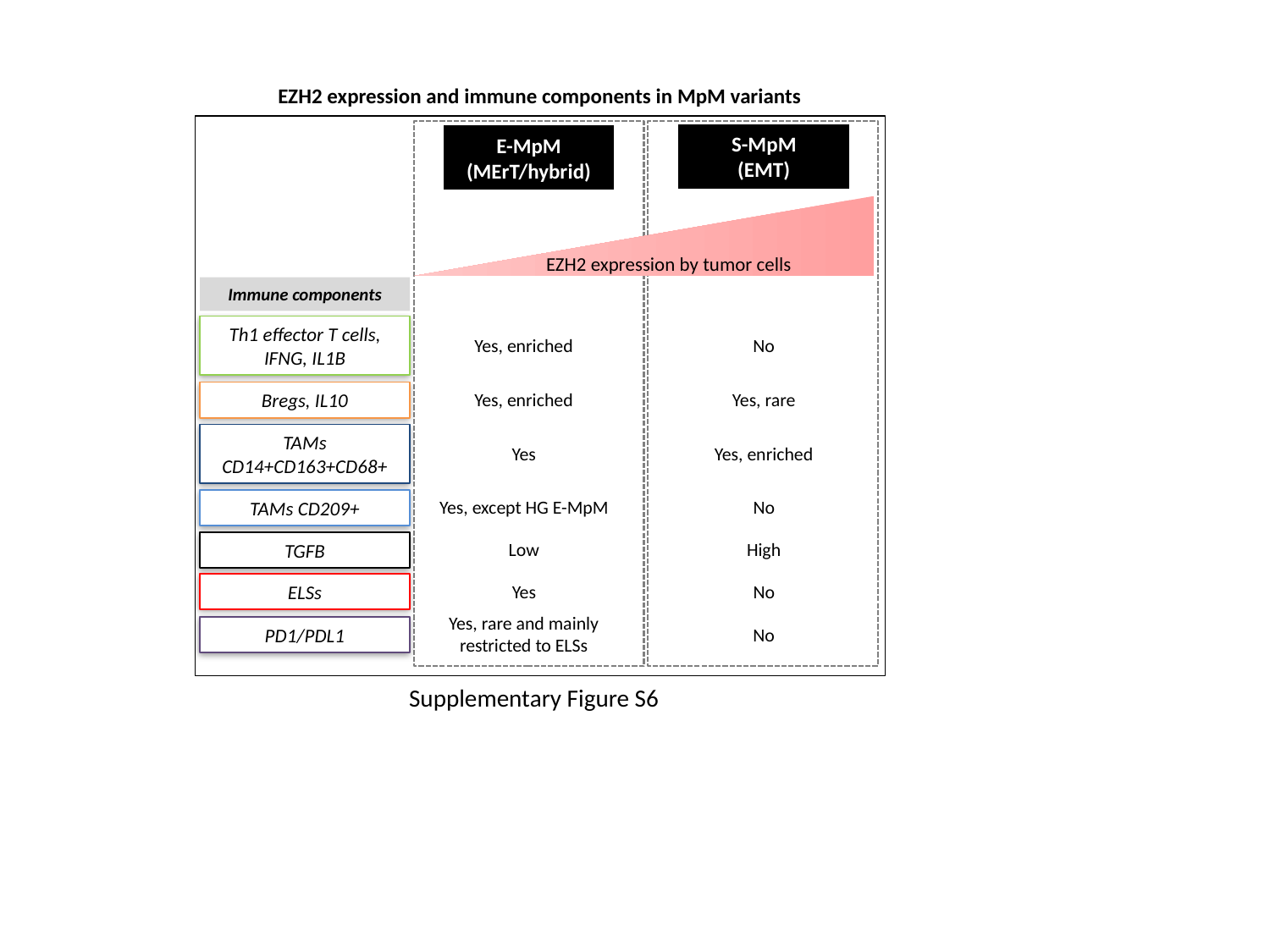

EZH2 expression and immune components in MpM variants
S-MpM
(EMT)
E-MpM
(MErT/hybrid)
EZH2 expression by tumor cells
Immune components
Th1 effector T cells, IFNG, IL1B
Yes, enriched
No
Bregs, IL10
Yes, enriched
Yes, rare
TAMs CD14+CD163+CD68+
Yes
Yes, enriched
TAMs CD209+
Yes, except HG E-MpM
No
TGFB
Low
High
ELSs
Yes
No
Yes, rare and mainly restricted to ELSs
PD1/PDL1
No
Supplementary Figure S6
